# A Deep Learning Approach for Managing Medical Consumable Materials in Intensive Care Units via Convolutional Neural Networks: Technical Proof-of-Concept Study

**DOI:** 10.2196/14806

**Published:** 2019-10-10

**Authors:** Arne Peine, Ahmed Hallawa, Oliver Schöffski, Guido Dartmann, Lejla Begic Fazlic, Anke Schmeink, Gernot Marx, Lukas Martin

**Affiliations:** 1 Department of Intensive Care Medicine and Intermediate Care University Hospital Rheinisch-Westfälische Technische Hochschule Aachen Aachen Germany; 2 Clinomic GmbH Aachen Germany; 3 Chair for Integrated Signal Processing Systems Rheinisch-Westfälische Technische Hochschule Aachen University Aachen Germany; 4 Chair of Health Management School of Business, Economics and Society Friedrich-Alexander-University Erlangen-Nürnberg Nürnberg Germany; 5 Research Area Distributed Systems Trier University of Applied Sciences Trier Germany; 6 Research Area Information Theory and Systematic Design of Communication Systems Rheinisch-Westfälische Technische Hochschule Aachen University Aachen Germany

**Keywords:** convolutional neural networks, deep learning, critical care, intensive care, image recognition, medical economics, medical consumables, artificial intelligence, machine learning

## Abstract

**Background:**

High numbers of consumable medical materials (eg, sterile needles and swabs) are used during the daily routine of intensive care units (ICUs) worldwide. Although medical consumables largely contribute to total ICU hospital expenditure, many hospitals do not track the individual use of materials. Current tracking solutions meeting the specific requirements of the medical environment, like barcodes or radio frequency identification, require specialized material preparation and high infrastructure investment. This impedes the accurate prediction of consumption, leads to high storage maintenance costs caused by large inventories, and hinders scientific work due to inaccurate documentation. Thus, new cost-effective and contactless methods for object detection are urgently needed.

**Objective:**

The goal of this work was to develop and evaluate a contactless visual recognition system for tracking medical consumable materials in ICUs using a deep learning approach on a distributed client-server architecture.

**Methods:**

We developed Consumabot, a novel client-server optical recognition system for medical consumables, based on the convolutional neural network model MobileNet implemented in Tensorflow. The software was designed to run on single-board computer platforms as a detection unit. The system was trained to recognize 20 different materials in the ICU, while 100 sample images of each consumable material were provided. We assessed the top-1 recognition rates in the context of different real-world ICU settings: materials presented to the system without visual obstruction, 50% covered materials, and scenarios of multiple items. We further performed an analysis of variance with repeated measures to quantify the effect of adverse real-world circumstances.

**Results:**

Consumabot reached a >99% reliability of recognition after about 60 steps of training and 150 steps of validation. A desirable low cross entropy of <0.03 was reached for the training set after about 100 iteration steps and after 170 steps for the validation set. The system showed a high top-1 mean recognition accuracy in a real-world scenario of 0.85 (SD 0.11) for objects presented to the system without visual obstruction. Recognition accuracy was lower, but still acceptable, in scenarios where the objects were 50% covered (*P*<.001; mean recognition accuracy 0.71; SD 0.13) or multiple objects of the target group were present (*P*=.01; mean recognition accuracy 0.78; SD 0.11), compared to a nonobstructed view. The approach met the criteria of absence of explicit labeling (eg, barcodes, radio frequency labeling) while maintaining a high standard for quality and hygiene with minimal consumption of resources (eg, cost, time, training, and computational power).

**Conclusions:**

Using a convolutional neural network architecture, Consumabot consistently achieved good results in the classification of consumables and thus is a feasible way to recognize and register medical consumables directly to a hospital’s electronic health record. The system shows limitations when the materials are partially covered, therefore identifying characteristics of the consumables are not presented to the system. Further development of the assessment in different medical circumstances is needed.

## Introduction

A large amount of medical materials are consumed in the daily routine of intensive care units (ICUs). It is estimated that 85% of their healthcare costs are captured by three cost blocks, namely staff, clinical support services, and consumable medical materials [1,2]. The latter include, for example, sterile disposable material (eg, venous catheters or scalpels), material for body care (eg, absorbent pads, disposable flaps), or small materials (eg, needles, swabs or spatulas). A study of the International Programme for Resource Use in Critical Care (IPOC) quantified the daily cost for disposables in four European countries between 139.5€ (152.9 United States Dollars [USD]) (104.9€ [115 USD]–177.2€ [194.2 USD]) and 29.6€ (32.4 USD) (17.5€ [19.2 USD]–59.7€ [65.4 USD]), for drugs and fluids between 183.3€ [200.9 USD] (150.6€ [165 USD]–217.4€ [238.3 USD]) and 65.3€ (71.6 USD) (42.2€ [46.2 USD]–91.5€ [100.3 USD]) per patient [1]. Materials are often stored centrally in a departmental location, such as a materials warehouse or a centralized room, from which only the daily required material is taken and stored in proximity to patients. This is particularly relevant for infectious patients, as possibly contaminated material may have to be disposed of for hygienic reasons when the patient is discharged.

Due to the complexity of an ICU treatment, the financing of ICUs is often based on a flat-rate reimbursement scheme [3], resulting in a fixed daily hospital reimbursement for each day on the unit, not taking into account the reason for admission, the disease, or the resulting expenditure. In this scheme, individual medical or nursing measures are only remunerated with an additional fee in special cases (eg, blood transfusions or very complex interventions). As the consumption of the above-mentioned material has also been financed from the reimbursed lump-sum payment, this means the consumption of disposable medical material can hardly be recorded on a patient-related basis. It is largely unknown how many materials are needed for a single patient with a specific disease, so it is therefore not possible to carry out analyses in this respect even though these questions are highly relevant in daily practice. This is particularly true for storage and investment, as suboptimal management results in unnecessarily high storage maintenance costs. Furthermore, from a scientific perspective, timely and accurate documentation of medical consumables is critical, as it is especially noticeable in daily scientific work when medical measures are not documented in a timely manner. For example, the administration of an infusion solution is usually not documented until several minutes after the start of the procedure [4]. This makes retrospective data analysis (eg, in the field of machine learning) considerably more difficult as action and reaction are often critically time-linked, thus making the scientific evaluation of measures and data analysis significantly more time-consuming [5].

In some hospitals, this problem is solved by scanning a material-specific barcode when a disposable material is used at the patient site, which enables patient-specific billing. However, this means each article needs to be marked with an individual code. The use of wireless radio-frequency identification (RFID) has been assessed in the circumstances of patient care, however this requires the installation of specialized reader infrastructure and related management systems [6]. However, applying the previous techniques for identification is impossible for a relevant part of the materials (such as swabs, needles) because of their physical structure or their low price in relation to the tagging technique. An intelligent, cost-effective solution is urgently needed for this problem, and the developed solution must suit the specific needs of the ICU with the following characteristics: (1) recognizes materials without explicit labelling (eg, barcodes, radio frequency labelling); (2) fulfills the high standards for quality and hygiene of ICUs (ideally minimizing touch-interactions); and (3) has minimal consumption of resources (eg, cost, time, training, and computational power) [7]. Most notably, the system must fulfill the high data protection requirements of the sensitive area of intensive care medicine.

The aim of this work is to develop and evaluate Consumabot, a novel client-server recognition system for medical consumable materials based on a convolutional neural network, as an approach to solve the above-mentioned challenges in the sector of intensive care medicine. We first described the technical background (hardware and machine learning), taking the specific limitations and challenges of the ICU sector into account. In a proof-of-concept study, we then evaluated the performance of the system in the adverse circumstances of a real ICU environment, assessing the feasibility of the application in a real-world hospital setting. 

## Methods

### General Layout

**Figure 1:**
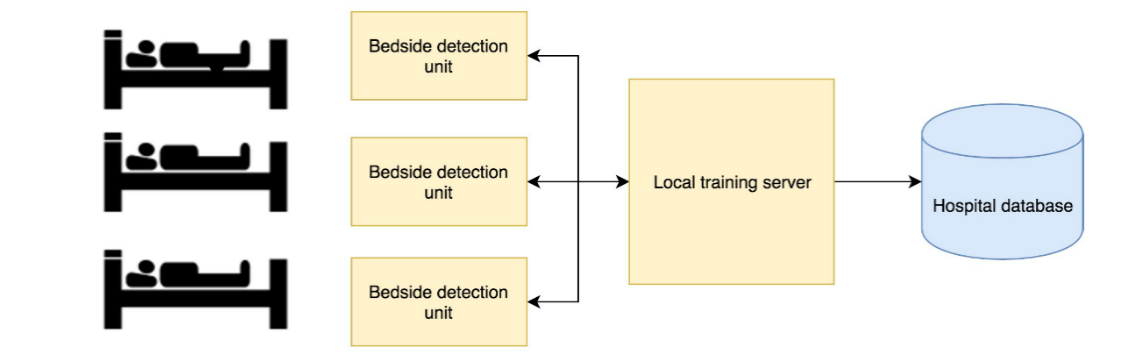
Client-server setup between bedside detection units, local training server, and hospital database of the Consumabot system.

We performed a thorough analysis on the technical, medical, and economic circumstances of ICUs and defined the specific requirements of the system. This included the identification of the need for a visual and contact-free recognition of the consumables, and for detection in proximity to the patient bed to facilitate assignment to the individual patient. Based on these considerations, the distributed concept of Consumabot was developed as follows: multiple low-cost detection units are located close to the patient bed, then these units are wirelessly connected to a local training server with high computational power for model training. This server has a direct connection to the hospital database and the electronic health record (EHR) backend (Figure 1).

### Hardware Setup

For the bedside detection units (clients), the commercially available single board computer Raspberry Pi was chosen as the hardware platform. Raspberry Pi is an inexpensive and widespread, small, single-board computer, initially developed to promote teaching of basic computer science in schools and in developing countries. The system architecture was developed at the University of Cambridge and is now being promoted by the charitable foundation the Raspberry Pi Foundation [8]. With low power consumption, no need for active cooling, and designed for continuous operation over several weeks, it is the ideal platform for the bedside detection units. Due to the popularity of the system, versatile extensions are available (eg, a powerful camera and hygienic housings). Indeed, Raspberry Pi has seen widespread use in different Internet of Things (IoT) applications in the healthcare sector [9-11].

**Figure 2:**
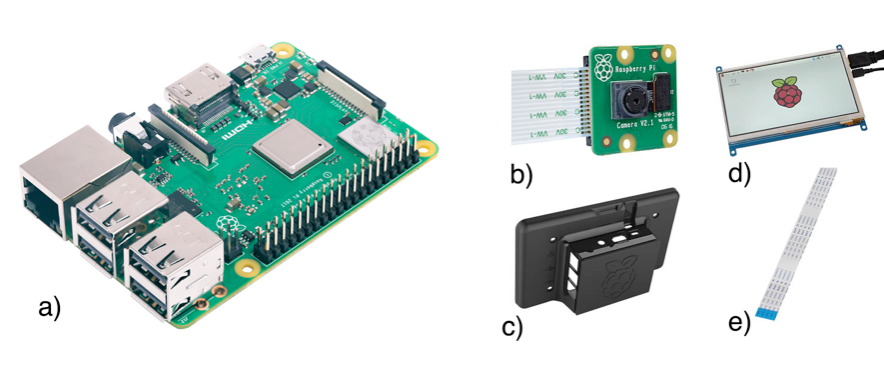
Hardware setup of the recognition module on the Raspberry Pi computational platform: a) Raspberry Pi 3 Model B; b) camera module version 2.1; c) Polyethylene housing; d) touch screen monitor module with 7 inch display diagonal; and e) Flat ribbon cable.

In this work, we specifically used the Raspberry Pi 3 Model B for the detection units (clients), equipped with a quad-core processor ARM Cortex A53, a network and wireless network card, and one gigabyte of random accessible memory. The official camera module of the Raspberry Pi Foundation version 2.1, with 8-megapixel resolution (Figure 2b), as well as a touch screen monitor module with 7-inch display for interaction with the software (Figure 2d) were used. The whole setup was installed into a stable polyethylene housing (Figure 2c), and the camera was positioned using flat ribbon cable (Figure 2e). The local recognition modules could not be used for the training of the neural network since the computational capabilities of the processors are too low, thus resulting in a long training time. 

Consequently, a more computationally capable training server was set up. This server was equipped with an Intel Xeon Gold 6140 processor with four dedicated processor cores, had 40 gigabytes of storage space on a single-state hard disk and had 320 gigabytes of storage space on a conventional magnetic hard disk for the resulting training data. Both the recognition module and the server used the open source operating system Linux in the Debian distribution [12].

### Training Setup and Machine Learning Scheme

The software backbone of Consumabot was developed in Python, a programming language often used in the field of machine learning. One major advantage of Python is the availability of a wide range of machine learning tools like *NumPy*, used in data preprocessing, *scikit-learn*, used for data mining, or *Keras* as a high-level neural network interface. For modelling the machine learning backend, we adapted the model of a convolutional neural network (CNN) [13]. The convoluted (folded) structure makes CNNs particularly suitable for processing visual information, especially in the fields of image recognition and classification [14-16]. However, manual development of a neural network is very time-consuming, so software frameworks in which essential mathematical and preprocessing steps have already been developed are often used. Consumabot uses the software library *TensorFlow*, a software framework that simplifies the programming of data stream–centered procedures [17], and several adapted programming code elements for retraining image classifiers were included into Consumabot’s source code [18]. Since the training of a full neural network is a complex and computationally intensive process, we applied a technique called transfer learning, a machine learning method where a model developed for a task is reused as the starting point for a model on a second task [19]. In transfer learning, basic processing image recognition steps, such as the recognition of edges, objects, and picture elements, are already trained in many iteration steps while the classification task is only assigned to the neural network in its final step, which is analogous to the training of an infant. However, it is important to note that the quality of the classification depends on the specificity of the training of the respective net. Thus, a neural network trained on images achieves better results in this domain than in the domain of something like natural language processing, resulting in the need to choose a task-specific, suited, pretrained network.

In the first step of the training the bottlenecks were generated, and they are the layer of the network that is located directly below the output layer [20]. Since each image is used several times, the bottlenecks do not change during the training as they can be created once and stored temporarily. In the second step, a set of random images from the training data set, with associated bottlenecks, were selected and placed in the output layer. The network-classified predictions are then compared with the correct classification, adjusting the weighting of the layers backwards (backpropagation) [21]. Classification accuracy and training progress were tracked with Tensorboard, a software designed for monitoring the training of neural networks [17,21].

### Classification and Feedback

The internal structure of the system must take the particularities of the ICU environment into account. The detection module's camera took a picture every second and stored it on a memory card. This image from the camera was presented to the trained model of the recognition unit, which then predicted the probabilities of the recognized objects. If anything other than an empty surface was detected then the recognized object with the greatest probability was selected and presented to the user. After pressing the *store in database* button on the screen, the material was then registered in the database of the training server. This resulted in an additional confirmation step of human classification, as only images confirmed to have been classified correctly were included in further training. To facilitate online learning, at regular intervals the stored images of correctly recognized materials were transferred as training data to the training server and the model of the neural network was trained. The resulting model (the retrained graph) was distributed among the recognition units, which enabled an improved recognition of the desired consumables. Finally, the database of the control server was used to further process the data for either analysis or optimization.

### On-Site Study

After finishing the training, we installed the hardware on an ICU within the University Hospital Aachen. Testing the system in real ICU conditions is obligatory due to the specific lighting and environmental conditions. In a test series, the 20 objects specified in Textbox 1 were classified using the camera of the recognition unit. Different rotations and orientations of the objects were chosen to correspond to the realistic field of application. To simulate the adverse circumstances of typical clinical workflow, we simulated a total of three scenarios: (1) scenario one, where the material was presented without any visual obstruction to the detection unit; (2) scenario two, where the material was 50% covered to simulate a visual obstruction during the routine clinical workflow; and (3) scenario three, where a secondary material (skin disinfection bottle) was present in the visual field while the material was presented without visual obstruction.

Each material was presented 10 times to the system. An object was classified as correct if it correctly appeared on the screen as the most probable classification (top-1 accuracy).

A full video of the hardware setup and training process can be found in Multimedia Appendix 1.

We performed a repeated measures, one-way analysis of variance (ANOVA) with Geisser-Greenhouse correction and a Tukey’s multiple comparisons test with individual variances computed for each comparison. This was performed to assess the effect of adverse conditions in scenario two and three in comparison to the nonobstructed view in scenario one. All calculations were performed using GraphPad Prism 8.1.2 (GraphPad Software Inc, San Diego).

**Selection of medical consumables.**

- Disposable bag valve mask
- Ampoule
- AuraOnce laryngeal mask
- Berotec inhalator
- Hand disinfection bottle
- Documentation sheet
- Boxed Dressings
- Packaged Gauze bandage
- Unpackaged Gauze bandages
- Gelafundin infusion solution
- Intravenous access orange
- Tube set for infusion solutions
- Intravenous access grey
- Braun sterile syringe
- Green Molinea protective pad
- White Protective pad
- Oxygen mask
- Oxygen tubing for mask
- Infusion solution Sterofundin
- Empty scenario (reference)

## Results

### Principal Findings

We trained the system in the context of a real ICU, taking special lighting conditions and other circumstances into account. We randomly chose a total of twenty common medical consumables from diverse categories with various sizes and formats to train the system on-site (Textbox 1). An empty scenario where no materials were present was provided to the system as a reference.

### Setup of the System and Training

**Figure 3:**
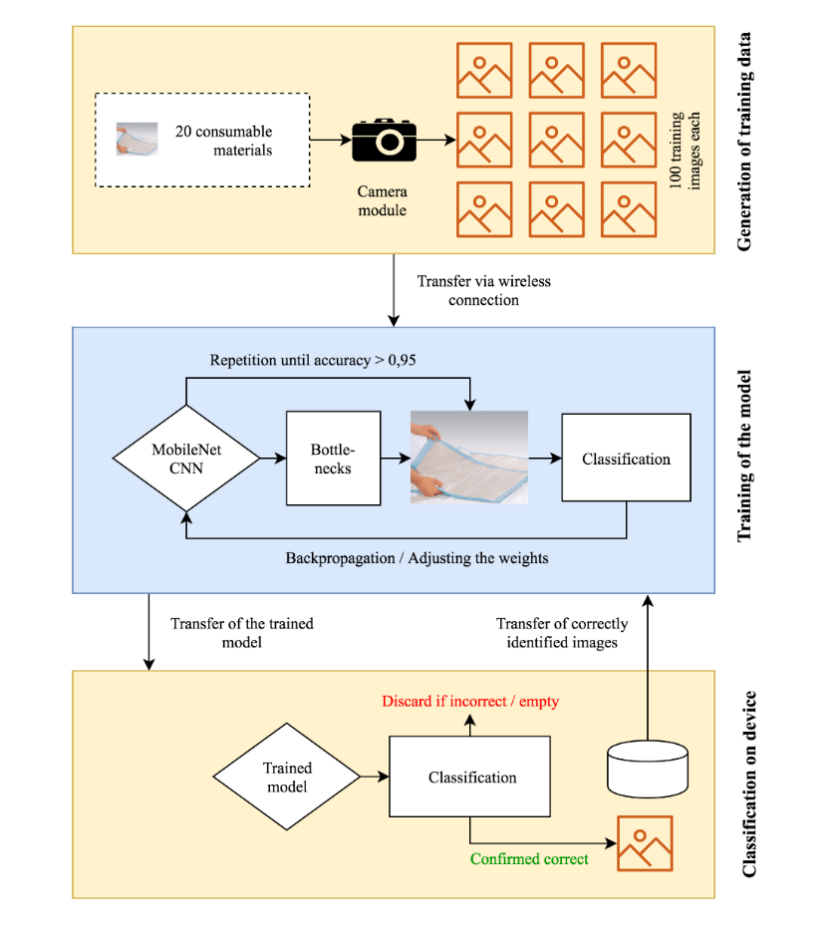
Overall setup of the Consumabot system. CNN: convolutional neural network.

Figure 3 shows the overall setup of Consumabot. The initial training was carried out using a newly developed data generation script, generating a series of 100 images of each medical consumable to be recognized. A total of 2000 images were generated. For training, we used 1800/2000 (90%) images, and 200 images were randomly picked from this training set for validation. Finally, the remaining 200/2000 (10%) were picked for testing. We ran the system for 500 epochs, or training steps (training, validation, and testing), each epoch consisting of 100 randomly chosen images per item.

The top layer of the CNN received a 1001-dimensional vector as input for each image, and we trained a softmax layer on top of this vector representation [22]. Assuming the softmax layer contains N labels, this corresponds to learning of 1001 × N model parameters corresponding to the learned weights and biases.

For choosing the appropriate network, we took the recognition accuracy of different convolutional neural networks into account. For this purpose, we used the top-1 score [23]. Briefly, in this process, the predicted class multinomial distribution (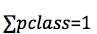) is obtained and compared to the appearance of the top classification as the target label (having the highest probability). The top-1 score is then computed as the times a predicted label matched the target label, divided by the number of data points evaluated. Selecting the correct model also needs to take the computational requirements into account, as the computational power of the recognition module is limited. Comparing MobileNet, AlexNet, GoogleNet and VGG16, we decided to apply a MobileNet, a class of efficient models for mobile and embedded vision apps, as a compromise between low requirements for computational power and high accuracy in image classification [24].

### Evaluation of Training Setup and Machine Learning Scheme

**Figure 4:**
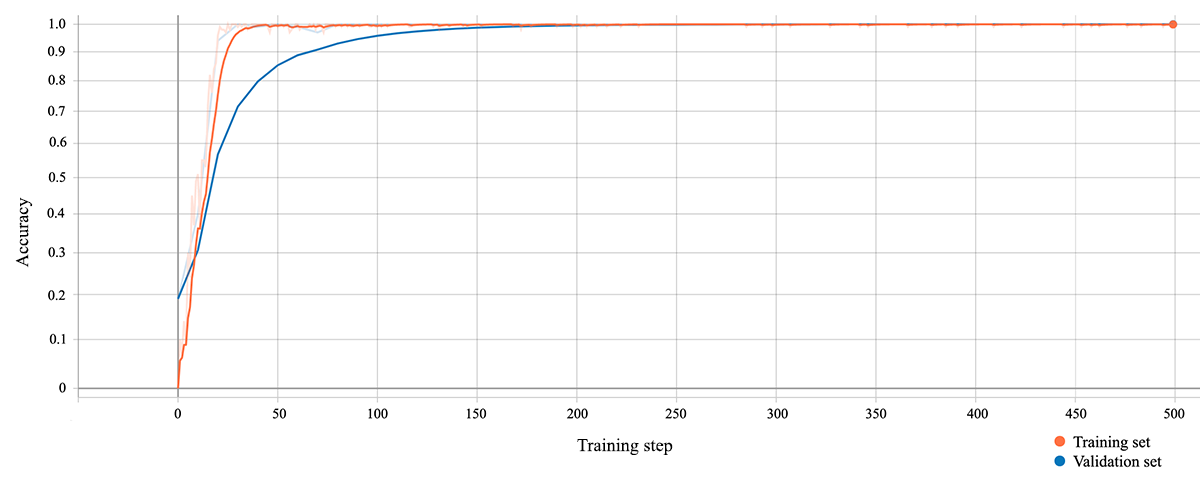
Accuracy of the model. The orange line represents the accuracy of correctly classified consumable material within the training set, while the blue line represents the accuracy of correctly classified consumable material within the validation set. A smoothing weight of 0.7 was applied and nonsmoothed curves are shown in pale orange and blue.

Figure 4 shows the performance of the learning algorithm on the training and validation datasets. We observed a >99% accuracy of the model prediction after 60 training steps and 150 validation steps, as defined by the relation of true positives over the total number of occurrences. The prediction accuracy approached asymptotically and remained there during the training phase, consequently showing a high prediction accuracy. Of note, there was no sign of overfitting [25], a phenomenon indicated by an increase or constancy in training accuracy while there is an observed decrease in validation accuracy. Thus, since our model did not show any indication of over-learning, the generalization of the output of the learned model is applicable. The smoothing linear filter is explained in Multimedia Appendix 2.

**Figure 5:**
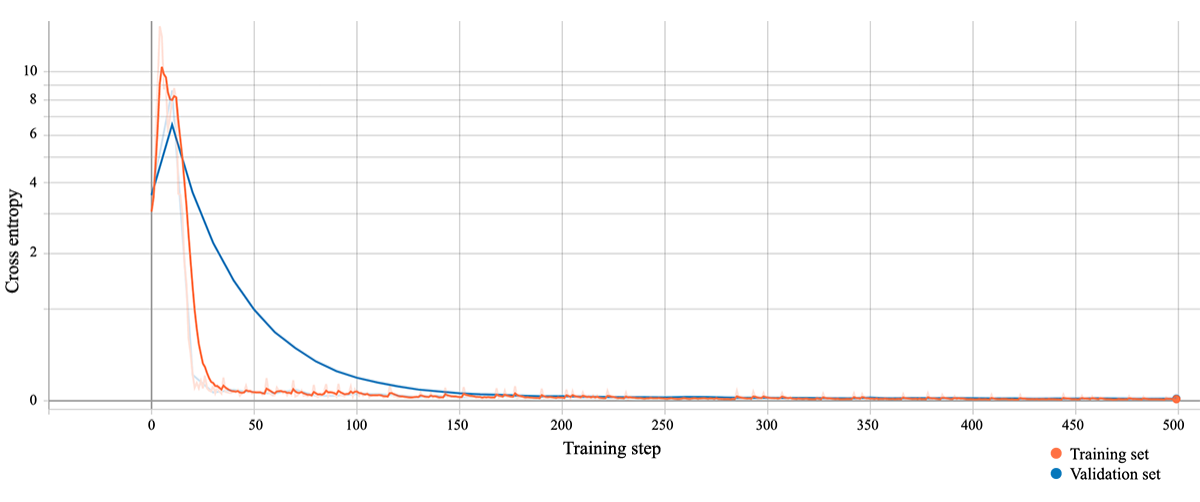
Cross-entropy of the model during training. The orange line represents the entropy of the training set, while the blue line represents the entropy of the validation set. A smoothing weight of 0.7 was applied, and nonsmoothed curves are shown in pale orange and blue.

Like all common machine learning techniques, the used model also requires a cost function that has to be minimized. We used cross-entropy as a cost function [26]: 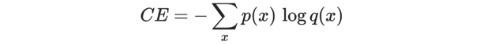


We applied this function to quantify the difference between the two probability distributions of the training and the validation set. As shown in Figure 5, our model revealed a desirable cross-entropy <0.03, with asymptotic stability after approximately 170 iteration steps in the validation set and 100 steps in the training set (Figure 5). As for Figure 4, the smoothing linear filter is explained in Multimedia Appendix 2.

The fully trained model (graph visualization, retrained graph, label list) is provided in Multimedia Appendices 3 and 4.

### On-Site Study

We then performed the on-site study at an ICU at the University Hospital Aachen, taking the particular lighting conditions and adverse circumstances due to clinical workflow into account. Each of the 20 consumable materials were presented ten times to the detection unit and were classified as correct if they appeared correctly on the screen with the highest associated probability (Table 1). The data generation for an entirely new consumable or for retraining, if a previously trained consumable material significantly changed its outer appearance, took approximately 100 seconds (1 second per picture, 100 pictures). We simulated adverse visual conditions as described in the methods section. For comparability reasons, no human feedback was included into the model training, and classification was based only on the first 100 training images. In Table 1, accuracy is provided in fractions of 1, as in a 0.7 recognition accuracy represents 70% (7/10) correct predictions.

**Table 1:** Top-1 recognition accuracy in the three scenarios.

Consumable material	Noncovered	Partially covered	Multiple materials
Bag valve mask	1	0.8	0.9
Ampoule	0.8	0.6	0.6
AuraOnce laryngeal mask	0.9	0.8	0.8
Berotec inhalator	0.7	0.8	0.7
Hand disinfection bottle	0.9	0.7	0.9
Documentation sheet	1	0.7	0.9
Boxed Dressings	0.8	0.7	0.7
Packaged Gauze bandage	0.8	0.7	0.8
Unpackaged Gauze bandage	1	0.9	0.8
Gelafundin infusion solution	0.8	0.7	0.8
Intravenous access orange	0.6	0.4	0.5
Tube set for infusion solutions	0.9	0.7	0.9
Intravenous access grey	0.8	0.6	0.7
Sterile syringe	0.9	0.7	0.8
Molinea protective pad green	0.8	0.8	0.7
White Protective pad	0.9	0.8	0.8
Oxygen mask	0.7	0.5	0.8
Oxygen tubing	0.9	0.6	0.9
Infusion solution Sterofundin	0.8	0.7	0.9
Empty scenario (reference)	1	1	0.8

**Figure 6:**
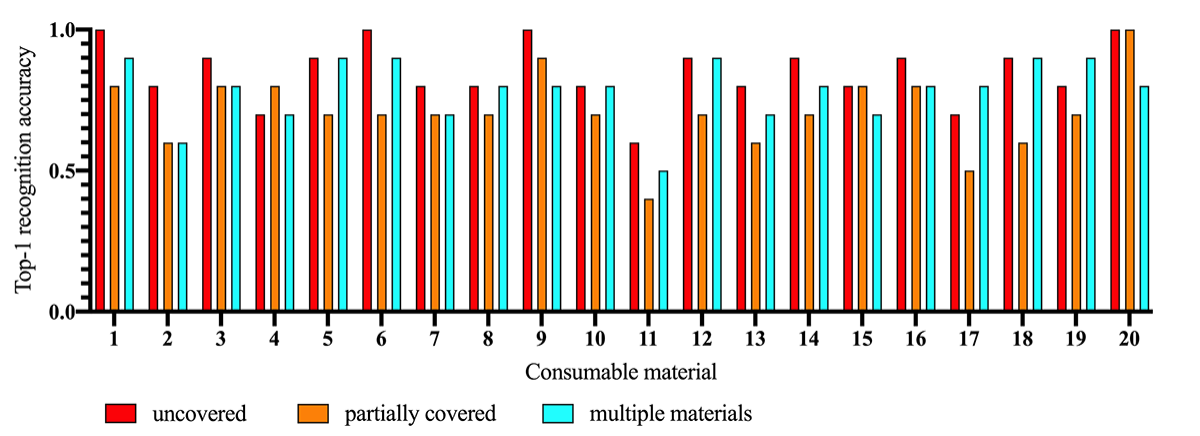
Results of the usability study in the context of a real ICU, real-world top-1 recognition accuracy of twenty sample materials. Top-1 recognition accuracy is provided in fractions of 1. Consumable materials: 1. AmbuBag (Disposable bag valve mask), 2. Ampoule, 3. AuraOnce laryngeal mask, 4. Berotec inhalator, 5. Hand disinfection bottle, 6. Documentation sheet, 7. Boxed Dressings, 8. Packaged gauze bandage, 9. Unpackaged gauze bandages, 10. Gelafundin infusion solution, 11. Intravenous access orange, 12. Tube set for infusion solutions, 13. Intravenous access grey, 14. Braun sterile syringe, 15. Molinea protective pad green, 16. White protective pad, 17. Oxygen Mask, 18. Oxygen tubing for mask, 19. Infusion solution Sterofundin, 20. Empty scenario (reference). ICU: intensive care unit.

In nonobstructed visual conditions, the model showed a good recognition performance, with a mean recognition accuracy of 0.85 (SD 0.11). Materials with large surface areas and many distinguishable visual features (eg, a disposable bag valve mask [mean recognition accuracy 1.0] or sterile syringes [mean recognition accuracy 0.9]) had particularly good detection rates. For materials only distinguishable by color (eg, intravenous [IV] accesses in different colors) Consumabot showed lower recognition accuracies for the grey IV access (mean 0.8) and the orange IV access (mean 0.6) (Figure 6, Table 1).

In a scenario where the surface area of the material was 50% covered, the system showed a lower, although still acceptable, mean recognition accuracy of 0.71 (SD 0.13). This was particularly true for materials with a small surface or with less distinguishable features (eg, for an oxygen tube), where the recognition accuracy dropped by 0.3 between when it was uncovered (mean 0.9) to when it was covered (mean 0.6).

Assessing the performance of the system in a scenario with multiple elements present in the scene resulted in a mean recognition accuracy of 0.78 (SD 0.11). For small elements compared to the secondary material present in a scene, this mostly resulted in a drop in recognition accuracy (eg, for a medication ampoule in the noncovered scenario [mean 0.8] versus a multiple element scenario [mean 0.6]).

We also performed an ANOVA with repeated measures to quantify the effect of the different scenarios, representing adverse real-world circumstances. Results of the ANOVA and other statistical analyses are given in Tables 2-4.

**Table 2:** Summary of the repeated measures ANOVA.

Test	F	*P* value	R^2^	Geisser-Greenhouse's epsilon
ANOVA^a^ summary	16.2	<.001	0.46	0.76
Matching effectiveness	4.89	<.001	0.57	—^b^

^a^ANOVA: analysis of variance

^b^Not applicable.

**Table 3:** Results of the Tukey's multiple comparisons test.

Comparisons	Mean difference	95% CI	Adjusted *P* value
Noncovered versus covered	0.14	0.08-0.2	<.001
Noncovered versus multiple	0.07	0.02-0.11	.001
Covered versus multiple	–0.075	–0.15 to 0.003	.06

**Table 4:** Detailed results of the repeated measures ANOVA.

Measures	Sum of squares	Degrees of freedom	Mean squares	F (DFn^a^, DFd^b^)	*P* value
Treatment (between columns)	0.20	2	0.1	F (1.5,29)=16	<.001
Individual (between rows)	0.56	19	0.03	F (19, 38)=4.9	<.001
Residual (random)	0.23	38	0.01	—^c^	—
Total	0.99	59	—	—	—

^a^DFn: degrees of freedom numerator

^b^DFd: degrees of freedom denominator

^c^Not applicable.

Independence of the observations among the groups, no sphericity, and a normal distribution were assumed for the analysis. The results of the performed ANOVA showed significant differences between the groups (F=16.2; *P*<.001; R^2^=0.46), and *post hoc* analyses with Tukey's multiple comparisons test showed a significant difference between the noncovered cohort and the partially covered cohort (*P*=.001; 95% CI 0.08-0.2). Further significant differences between the noncovered scenario and the scenario with multiple consumables were also observed (*P*=.001; 95% CI 0.02-0.11), however, the differences between the noncovered group and multiple consumables were not statistically significant (*P*=.06).

## Discussion

In this work we developed and evaluated Consumabot, a novel contactless visual recognition system for tracking medical consumable materials in ICUs using a deep learning approach on a distributed client-server architecture. In our proof-of-concept study in the context of a real ICU environment, we observed a high classificatory performance of the system for a selection of medical consumables, thus confirming its wide applicability in a real-world hospital setting.

Building on the foundation of fundamental mathematical research and technical progress, machine learning technologies today have the potential to drive ICUs towards a more sustainable, data-driven environment. In particular, contributions from other scientific disciplines, such as biology and engineering, have led to significant breakthroughs in quality and availability of neural networks, thus forming the backbone of Consumabot. The development of software for processing complex visual information is no longer a task requiring specialized hardware and software, as even the training of a complex neural network without specialist knowledge is possible now. This enables researchers and medical professionals alike to expand the use of artificial intelligence beyond today's commercial applications, such as in the fields of natural language processing [27,28], or intention, or pattern analysis [29], within constantly growing data volumes.

In this work we demonstrated the feasibility of the application of an on-device, deep learning–based computational platform for optical material recognition in the context of an ICU. Using a convolutional neural network infrastructure, the system Consumabot consistently achieved good results in the classification of consumables and thus is a feasible way to directly recognize and register medical consumables to a hospital’s EHR system. Choosing a transfer learning technique based on MobileNet assured a fast training time while keeping steadily high recognition rates, achieving an optimal compromise of high accuracy and low computational requirements while maintaining a moderate model size. Using an optical recognition approach takes the specific conditions of the ICU into account, such as the need for a low maintenance, hygienic, contact-free solution. The use of MobileNet allowed us to apply Consumabot to the inexpensive, computationally weak, Raspberry Pi platform, while maintaining acceptable recognition speed. This confirmed the feasibility of use of the Raspberry Pi platform in healthcare, as described in multiple earlier works [11,30,31]. The upcoming increase in computational power of single-board computers could make the distributed client-server structure of the system unnecessary, as training could take place directly on the recognition units. This will enable its direct use in environments where no network connectivity is available (eg, rural areas), potentially facilitating scientific research in less developed medical infrastructure and healthcare systems. Thus, we believe that Consumabot will ultimately enable hospitals to reduce costs associated with consumable materials and consequently let them spend their resources on higher quality care (eg, by employing additional medical personnel).

Nevertheless, the conducted on-site study showed potential for optimization, particularly for standard medical consumables (eg, venous accesses of different sizes) since they did not show fully satisfactory recognition rates. This occurred if the distinguishing features were not clearly visible, partially covered, or multiple consumables were present in the scene. To solve this problem, a user training course with a note that identifying features must be clearly presented is recommended. Further, the model performance is likely to increase with added training data during daily use in the ICU due to the implemented feedback mechanism of Consumabot. Further development of the software and assessment of its performance with larger sets of medical consumables is desirable. The presentation of an object unknown to the detection unit results in a prediction with low confidence. In our prototype we display this confidence factor (see Multimedia Appendix 1) to the user. When using Consumabot in scenarios where many objects are unknown to the system (eg, if the system will only be used for detection of certain objects), the software should be adapted to only display predictions above a certain confidence threshold. In addition, the performance of multiple detection units running in parallel needs to be assessed as potential conflicts in the distribution of the model could occur.

Overall, though, the results were satisfactory enough to promote the further use and development of Consumabot in practice and research. The system fulfilled the requirements for recognizing materials without explicit labelling while maintaining the standard for quality and hygiene of ICUs. The system will make retrospective data analysis (eg, in the field of machine learning) considerably easier and enable time-critical research with direct correlation between action and reaction. The prototype’s capabilities could potentially be enhanced by the integration of visual, multi-object detection algorithms, thus enabling it to detect a multitude of objects in parallel. Further, the need for tactile manual confirmation could be reduced by the integration of a microphone array to enable voice commands. The full source code of the detection unit, the pretrained model, and the training script have been released under the open source license Apache Version 2.0, January 2004 [32], and detailed assembly instructions have been released with the manuscript to encourage and enable other researchers to contribute to the development of the system and assess usability and feasibility in other use cases without increasing the financial burden of ICU patients [33].

## Appendix

Multimedia Appendix 1 Demonstration video of the full Consumabot setup.

Multimedia Appendix 2 Applied smoothing algorithm in Python.

Multimedia Appendix 3 Retrained Tensorflow graph for materials used in experiment.

Multimedia Appendix 4 Labels for Tensorflow model.

## Abbreviations

ANOVA: analysis of variance
CNN: convolutional neural network
EHR: electronic health record
ICU: intensive care unit
IoT: Internet of Things
IPOC: International Programme for Resource Use in Critical Care
IV: intravenous
RFID: radio-frequency identification
USD: United States Dollar
