# Supplementary material for: A Deep Learning Approach for Managing Medical Consumable Materials in Intensive Care Units via Convolutional Neural Networks: Technical Proof-of-Concept Study
Source: JMIR Med Inform. 2019 Oct 10;7(4):e14806. doi: 10.2196/14806 (PMC6819012; doi:10.2196/14806)
Supplement: Multimedia Appendix 2 [file medinform_v7i4e14806_app2.pdf]

```
def smooth(scalars, weight):  # Weight between 0 and 1, 0.7 in Consumabot case
    last = scalars[0]  # First value in the plot (first timestep)
    smoothed = list()
    for point in scalars:
        smoothed_val = last * weight + (1 - weight) * point  # Calculate smoothed value
        smoothed.append(smoothed_val)                        # Save it
        last = smoothed_val                                  # Anchor the last smoothed value

    return smoothed
```
