# Supplementary material for: A Deep Learning Approach for Managing Medical Consumable Materials in Intensive Care Units via Convolutional Neural Networks: Technical Proof-of-Concept Study
Source: JMIR Med Inform. 2019 Oct 10;7(4):e14806. doi: 10.2196/14806 (PMC6819012; doi:10.2196/14806)
Supplement: Multimedia Appendix 4 [file medinform_v7i4e14806_app4.pdf]

Top-1 recognition accuracy is provided in fractions of 1. Consumable materials: 1. AmbuBag (Disposable bag valve mask), 2. Ampoule, 3. bag valve mask, 4. Berotec inhalator, 5. Hand disinfection bottle, 6. Documentation sheet , 7.Dressings boxed, 8. Gauze bandage packaged, 9. Gauze bandages unpackaged, 10. Gelafundin infusion solution, 11. Intravenous access orange, 12. Tube set for infusion solutions, 13. Intravenous access grey, 14. Braun sterile syringe, 15. Molinea protective pad green, 16. Protective pad white, 17. O2 Mask, 18. O2 tubing for mask, 19. Infusion solution Sterofundin, 20. Empty scenario (reference)
